# Effectiveness of septoplasty versus non-surgical management for nasal obstruction due to a deviated nasal septum in adults: study protocol for a randomized controlled trial

**DOI:** 10.1186/s13063-015-1031-4

**Published:** 2015-11-04

**Authors:** M. M. H. T. van Egmond, M. M. Rovers, C. T. M. Hendriks, N. van Heerbeek

**Affiliations:** Department of Otorhinolaryngology, Radboud university medical center, Radboud Institute for Health Sciences, route 377, PO Box 9101, 6500 HB Nijmegen, the Netherlands; Department of Operating Rooms, Radboud university medical center, Radboud Institute for Health Sciences, route 715, PO Box 9101, 6500 HB Nijmegen, the Netherlands

**Keywords:** Septoplasty, Deviated nasal septum, Nasal obstruction, Quality of life, Rhinomanometry, Cost-effectiveness

## Abstract

**Background:**

Septoplasty, i.e., surgical correction of the deviated nasal septum, is the most common ear, nose and throat (ENT) operation in adults. Currently the main indication to perform septoplasty is nasal obstruction. However, the effectiveness of septoplasty for nasal obstruction in adults with a deviated nasal septum remains uncertain. Scientific evidence is scarce and inconclusive, and internationally accepted guidelines are lacking. Moreover, there is no consensus on whether or not septoplasty should be combined with concurrent turbinate surgery. The objective of the current ongoing trial is to study the effectiveness of septoplasty (with or without concurrent turbinate surgery) as compared to non-surgical management for nasal obstruction in adults with a deviated nasal septum, both in terms of subjective (health-related quality of life) as well as objective (nasal patency) outcome measures.

**Methods/Design:**

The study is designed as a pragmatic, multicenter, parallel-group, randomized controlled trial. A total of 200 adults will be enrolled with nasal obstruction based on a deviated nasal septum and an indication for septoplasty according to current medical practice in the Netherlands. Participants will be randomized to either septoplasty (with or without concurrent turbinate surgery as originally indicated by the otorhinolaryngologist) or a non-surgical watchful waiting strategy. Follow-up visits will be scheduled at 0, 3, 6, 12, and 24 months. During each follow-up visit, health-related quality of life questionnaires will be administered and measurements of four-phase rhinomanometry and peak nasal inspiratory flow will be performed. Costs will be studied using a patient-based diary. Effects of septoplasty on health-related quality of life (primary outcome) and nasal patency will be calculated as mean differences with 95 % confidence intervals. Subgroup analyses according to gender, age, and severity of the septal deviation will be performed. All analyses will be performed on an intention-to-treat basis.

**Discussion:**

With the results of this study we aim to contribute to the development of evidence-based guidelines regarding indications for septoplasty.

**Trial registration:**

Nederlands Trial Register/Dutch Trial Registry (www.trialregister.nl), trial identifying number: NTR3868. Registered on 21 February 2013.

**Electronic supplementary material:**

The online version of this article (doi:10.1186/s13063-015-1031-4) contains supplementary material, which is available to authorized users.

## Background

### Background and rationale

#### General introduction

Septoplasty is a surgical procedure that aims to straighten the deviated nasal septum. Septoplasty should not be confused with septorhinoplasty, which is a surgical procedure that addresses both the nasal septum as well as other structures of the nose: e.g., alar and triangular cartilages and nasal bones, most often to improve the appearance of the nose. Surgical correction of the deviated nasal septum is the most common ear, nose and throat (ENT) operation in adults [1]. Annual septoplasty rates, however, differ between countries. More than 20,000 septoplasties, i.e., 3.8 septoplasties per 10,000 inhabitants, were performed in England between 2012 and 2013 [2]. In the Netherlands, 10,000 septoplasties, i.e., 6.0 septoplasties per 10,000 inhabitants, were performed as a single procedure or in combination with turbinate surgery in 2010 [3].

#### Mechanisms

Currently, the main indication to perform septoplasty is nasal obstruction. Nasal obstruction is commonly defined as patient discomfort manifested as a sensation of insufficient airflow through the nose [4]. The etiology of nasal obstruction is generally divided into mucosal and anatomical causes. Nasal septal deviation is the most common anatomical cause of nasal obstruction [5]. Still, underlying pathogenesis is often multifactorial. For example, nasal septal deviation is commonly accompanied by compensatory mucosal hypertrophy of the contralateral turbinate [6]. This counterbalanced mechanism is assumed to protect the more patent nasal side from the drying and crusting effects of excess airflow [7]. Compensatory hypertrophy can occur in both the inferior as well as the middle turbinate on the side contralateral to nasal septal deviation [8]. It has been shown that turbinate enlargement not only comprises mucosal elements, but may also involve the conchal bone [6]. Since these changes may not be spontaneously reversible, they sometimes need to be corrected in conjunction with septal surgery to prevent nasal obstruction on the non-deviating side postoperatively [9, 10]. By straightening the deviated nasal septum and performing concurrent turbinate surgery, nasal passages are assumed to widen and as a result nasal breathing is thought to improve.

#### Existing knowledge

At present the effectiveness of septoplasty in adults with nasal obstruction and a deviated nasal septum remains uncertain. Objective measurements do not always correlate with subjective measures to evaluate the effectiveness of septoplasty [11]. Furthermore, additional benefits of concurrent turbinate surgery are unknown, and indications and techniques applied vary considerably [12, 13]. Remarkably, the literature shows a prevalence of nasal septal deviation of up to 80 %, whereas only a minority of subjects suffers from nasal obstruction [14]. More important in this respect, is the fact that scientific publications on the effects of septoplasty are scarce and inconclusive, i.e., there is little hard evidence that this procedure provides any benefit to the patient [14].

As requested by our grant provider, we performed a computerized literature search in PubMed, Cochrane Library, Current Controlled Trials, ClinicalTrials.gov, Database of Abstracts of Reviews of Effects (DARE), National Health Service Economic Evaluation Database (NHS EED), and Health Technology Assessment (HTA) Database, for systematic reviews or randomized trials on the effectiveness of septoplasty. Studies were included that met the following criteria: a) randomized trials or systematic reviews investigating the effectiveness of septoplasty; b) the control group received non-surgical management; and c) clinically relevant outcomes were reported: e.g., quality of life or rhinomanometry. Two reviewers (NvH, MR) independently assessed eligibility of studies. To ensure completeness, we did not use any quality assessments to select within the identified randomized trials. Information on patient characteristics (P), interventions (I), the contrast between interventions (C) and outcome measures (O) were extracted from all included studies. Initially, 287 studies were identified with our search strategy. All these studies, however, had to be excluded since they either studied other interventions (e.g., peri-operative care, postoperative care, or interventions like nasal packages or various forms of analgesia), or did not report on a randomized controlled trial. Besides, no data were found regarding cost-effectiveness of septoplasty versus watchful waiting in patients with a deviated nasal septum. Nonetheless, we identified a systematic review that showed evidence of a change in nasal airway patency in observational studies, in which pre-operative and postoperative results were compared [15]. Furthermore, we actively searched for observational studies that reported on the effect of septoplasty on health-related quality of life. We identified five observational studies that all compared pre-operative and post-operative health-related quality of life scores [16–20]. In all studies health-related quality of life increased postoperatively, suggesting the improvement to be caused by septoplasty. Since all available evidence is based on observational studies, the risk of bias is high, i.e., the reported beneficial effects could also be explained by other factors, such as natural history (no study reported on the natural course, i.e., the effect of watchful waiting), or extraneous effects (in many patients additional interventions were performed). Due to these biases the suggested effect of septoplasty might be overestimated. In short, the lack of randomized controlled trials precludes a definite conclusion concerning the effectiveness of septoplasty.

#### Need for a trial

Recently, the benefits of septoplasty have been questioned, with one of the main concerns being that possible gains are mainly subjective. In 2010, the professional association of UK otorhinolaryngologists published a position paper expressing their concern that some hospital administrations were suggesting the abolition or severe restriction of septal surgery, because of doubts over its benefits [21]. The Dutch ENT Society also acknowledged the lack of evidence for septoplasty as one the most important knowledge gaps in current otorhinolaryngology for which a randomized controlled trial is warranted [22].

### Objective

The objective of the current ongoing trial is to study the effectiveness of septoplasty (with or without concurrent turbinate surgery) as compared to watchful waiting for nasal obstruction due to a deviated nasal septum in adults, both in terms of subjective (health-related quality of life) as well as objective (nasal patency) outcome measures.

## Methods/Design

### Trial design

The septumtrial is designed as a pragmatic, multicenter, parallel-group, two-arm randomized controlled trial.

### Study setting

Patient recruitment is currently being conducted at otorhinolaryngology outpatient clinics in three tertiary referral hospitals and eighteen secondary referral hospitals spread over the Netherlands. A multicenter approach was chosen to increase chances of obtaining the desired power and to assure representativeness of our sample to the target population.

### Participants

#### Inclusion criteria

Patients eligible for the trial must comply with all of the following at randomization:

1. Be age 18 years or above;
2. Provide written informed consent;
3. Have an indication for septoplasty with or without concurrent turbinate surgery according to current medical practice, i.e., symptomatic impairment of the nasal passage due to nasal septal deviation.

In all patients internal exam of the nose should clearly document that the nasal septal deviation is causing a mechanical nasal airway obstruction and that the deviation is the primary contributing factor of obstructed nasal breathing.

#### Exclusion criteria

Patients selected for septoplasty because of nasal septal perforation, patients with a history of previous nasal septal surgery, as well as patients selected for septoplasty as part of a cosmetic rhinoplasty procedure, will be excluded. Patients with untreated allergic rhinitis or allergic rhinitis unresponsive to medical treatment will also be excluded. In Dutch clinical practice most patients are selected for septoplasty because of symptomatic impairment of the nasal passage due to nasal septal deviation. However, many patients will have concurrent complaints, such as impairment of normal sinus drainage, sleep disorders, or headaches. These patients can be included if the concurrent complaints comprise secondary indications. In cases where these complaints are the main indication for septoplasty, patients will also be excluded, as will cleft lip and/or palate patients.

### Interventions

#### Interventions

Included patients will be randomly assigned to either septoplasty (with or without concurrent turbinate surgery) performed within 6 to 8 weeks after randomization, or initial non-surgical management. Although septoplasty is often performed with concurrent surgery for compensatory turbinate hypertrophy, the additional benefits are questioned [12, 13]. In this trial it is allowed to combine septoplasty with concurrent turbinate surgery, which will be accounted for in our subgroup analyses. Septoplasty and concurrent turbinate surgery both need to be performed according to current medical practice. Several techniques for performing turbinate surgery are available. Where applicable, we will register which technique has been used in each trial patient. Non-surgical management includes watchful waiting and medical treatment such as steroids, antibiotics (intermittent and long-term), antihistamines, and analgesics. Since the benefits of septoplasty have never been sufficiently established, the selection of non-surgical management as comparator is justified.

#### Modifications

If they wish to do so, subjects can leave the study at any time for any reason without any consequences. Additionally, the investigator can decide to withdraw a subject from the study for urgent medical reasons. Subjects in the watchful waiting group may need to undergo surgery because of persisting complaints. As in all surgical trials, we expect about 30 % of patients to switch from watchful waiting to surgery. Patients wishing to cross-over from watchful waiting to surgery will be monitored for the complete follow-up period.

#### Concomitant care

Because of the pragmatic nature of the septumtrial, all patients in the septoplasty group as well as the watchful waiting group are allowed to use any kind of escape medication that is freely available (e.g., over-the-counter medication such as paracetamol, ibuprofen, normal saline nasal spray or drops, xylometazoline nasal spray or drops) or prescribed by their general practitioner or otorhinolaryngologist (such as corticosteroid nasal sprays, antibiotics, NSAIDs). All medication used as well as all additional doctor’s visits will be recorded in a patient-based diary to be able to calculate costs of these additional expenses.

### Outcomes

#### Primary outcome measure

Primary outcome measure will be the difference between both treatment arms in health-related quality of life, measured using the Glasgow Benefit Inventory (GBI) and Glasgow Health Status Inventory (GHSI) questionnaires, at the primary time point of 1 year after inclusion. While the GHSI gives a general measure of health status of a person at any specific time, the GBI is maximally sensitive to a change in health status caused by a specific event (e.g., surgical intervention) and can be administered postoperatively to measure the effect of the intervention on a patient’s quality of life [23]. Health-related quality of life was chosen as primary outcome measure, since septoplasty aims at improving nasal symptoms rather than merely straightening the nasal septum. Due to the dual chamber design of the nose, clinical practice shows no clear correlation between objective (e.g., nasal patency) and subjective results of septoplasty (e.g., patient sensation of airflow through the nose) [11]. Moreover, nasal septal deviation with concurrent reduction of nasal passage can be observed in asymptomatic subjects, while other subjects with comparable deviations may suffer from severe complaints. Apparently, what matters is not the nasal passage itself, but the effect the nasal passage has on someone’s well-being and daily life. Using quality of life measures in clinical practice ensures that treatment and evaluation focus on the patient rather than the disease. In addition, by using quality of life as primary outcome measure, we facilitate the comparison of treatment effects resulting from septoplasty with treatment effects of other procedures.

#### Secondary outcome measures

Secondary outcome measures comprise:

1. Other health-related quality of life measures, i.e., the EuroQol-5 Dimensions-3 Levels (EQ-5D-3 L), the Sino-Nasal Outcome Test-22 (SNOT-22), and the Nasal Obstruction Symptom Evaluation scale (NOSE);
2. Nasal patency as measured with four-phase rhinomanometry and peak nasal inspiratory flow;
3. Complications and potential side effects;
4. Cost-effectiveness.

The EQ-5D-3 L consists of a descriptive part and a visual analogue scale (VAS). The descriptive part comprises the following five dimensions: mobility, self-care, usual activities, pain/discomfort, and anxiety/depression. The VAS records the respondent’s self-rated health on a vertical scale ranging from 0 (worst imaginable health state) to 100 (best imaginable health state) [24]. The SNOT-22 is developed as a patient-reported outcome measure to use in chronic rhinosinusitis with or without nasal polyposis and covers a broad range of health and health-related quality of life problems, including physical problems, functional limitations, and emotional consequences [25, 26]. The NOSE is developed to assess nasal obstruction and is validated in adults undergoing septoplasty [16, 27].

The physician researcher, who performs four-phase rhinomanometry and peak nasal inspiratory flow measurements, has been trained in the use of both devices. The rhinomanometer was produced by the RhinoLab GmbH, Freiburg im Breisgau, Germany, and supplied by Dos Medical BV, ‘s-Hertogenbosch, the Netherlands. The inspiratory flow meter was produced by Clement Clarke International, Essex, United Kingdom, and supplied by PitMedical BV, Enschede, the Netherlands.

### Participant timeline

**Fig. 1:**
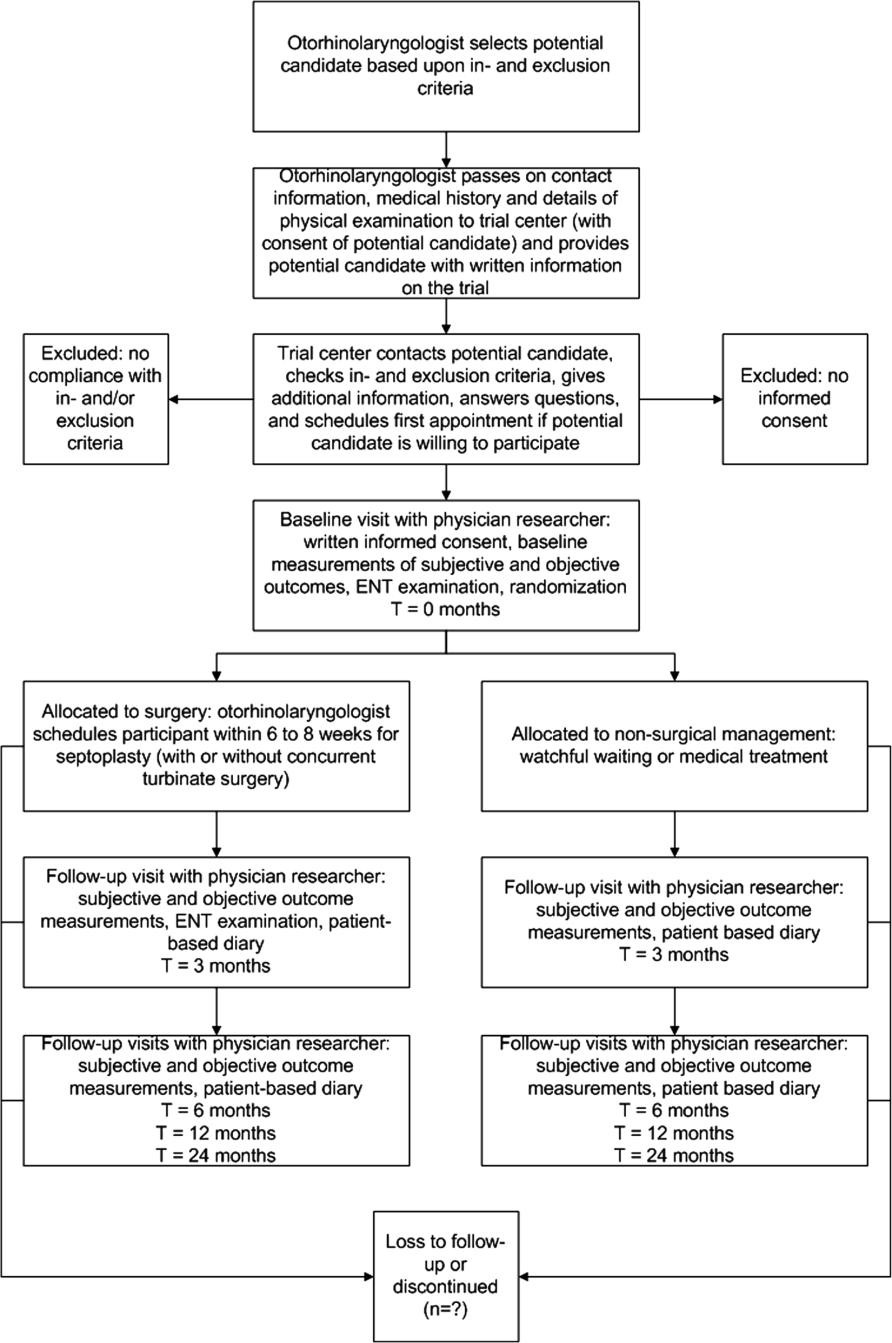
Flow-chart of participant timeline

With their informed consent, contact details of potential candidates will be communicated to the trial center by otorhinolaryngologists in the participating hospitals. Subsequently, patients will be contacted by phone by one of the researchers. The researcher will provide detailed information concerning the trial, answer questions, and check inclusion and exclusion criteria. When the patient agrees to participate, a baseline visit will be scheduled. At this visit, informed consent documents will be signed and baseline measurements will be performed, consisting of generic and disease-specific health-related quality of life questionnaires, disease-specific symptom scores, an ENT examination including anterior rhinoscopy and nasal endoscopy, four-phase rhinomanometry, and peak nasal inspiratory flow measurements^1^. Subsequently, the patient will be randomized to either septoplasty, with or without concurrent turbinate surgery as originally indicated by the otorhinolaryngologist, or a non-surgical watchful waiting strategy. The patient’s otorhinolaryngologist will be notified of the result of randomization. If the patient is assigned to the surgical group, septoplasty is scheduled by the otorhinolaryngologist within 6 to 8 weeks. A detailed and standardized operation report will be kept. During the study, patients and otorhinolaryngologists will be encouraged to manage nasal obstruction according to their regular practice. If the medical condition of a patient warrants attention during follow-up, the study physician will refer the patient to his or her local otorhinolaryngologist for further management. If the otorhinolaryngologist decides to perform septoplasty on a patient in the watchful waiting group (because of severe complaints persisting under non-surgical management), this patient will be classified as cross-over and monitored as planned. All included patients will be invited for follow-up visits at 3 months, 6 months, 12 months, and 24 months. During these four follow-up visits, the same questionnaires and measurements of the baseline visit are scheduled. Patients will be instructed to record health-related costs in a patient-based diary, which will be handed in and checked during each follow-up visit. Figure 1 shows a flowchart of our trial.

### Sample size

Calculations of group size are based on a clinically relevant improvement on the Glasgow scale of 10 (standard deviation 15). Assuming a quality of life of 70 in the non-surgical group, and taking an alpha of 0.05 and a power of 0.90, each treatment arm should include at least 48 patients. We also performed a power calculation for the objective rhinomanometry measurements. The mean value of four-phase rhinomanometry in patients is about 2.6 Pa/cm^3^.s^-1^ (standard deviation 2). Since the mean value in asymptomatic patients is considered to be about 1.5 Pa/cm^3^.s^-1^, we assume an improvement of 1 Pa/cm^3^.s^-1^ to be clinically relevant. Taking an alpha of 0.05 and a power of 0.90, each treatment arm should include at least 70 patients. To allow for subgroup analyses according to gender, age, and severity of the septal deviation, we will include at least 100 adults in each treatment arm.

### Recruitment

Otorhinolaryngologists in 21 Dutch hospitals are involved in patient recruitment. Both the support of the Dutch ENT Society, as well as the collaboration with a group of dedicated otorhinolaryngologists set up during previous studies, will help us to include sufficient patients in the trial. Screening and recruitment will continue until the target population (*n* = 200) is achieved.

### Allocation

#### Sequence generation

Participants will be randomly assigned to either septoplasty or non-surgical management. We will use a computerized minimization strategy, i.e., a method of ensuring balance between prognostic factors in small samples [28, 29]. Factors that will be taken into account are severity of the septal deviation, age, gender, and hospital in which the patient is treated.

#### Concealment mechanism

Allocation concealment will be ensured, as the result of randomization will not be released until the patient has been recruited into the trial, formal informed consent has been obtained, and all baseline measurements have been completed.

#### Implementation

Randomization will be performed by the study physician during the first study visit, after all baseline measurements have been completed.

### Blinding

Trial participants, researchers and otorhinolaryngologists will not be blinded after assignment to septoplasty or non-surgical management. As for trial participants and their otorhinolaryngologists, blinding is not possible since it would be unethical to propose sham surgery in the control group. Moreover, blinding of researchers is not needed since they are only involved in measuring objective outcomes.

### Participate retention

Once a subject is enrolled and randomized, the researchers will make every reasonable effort to follow the subject for the entire study period. All study visits will take place at the hospital in which the patient is receiving treatment. Study visits will be scheduled in agreement with subjects. Moreover, participants will receive a reminder before every study visit. Questionnaires will be administered digitally. In this way, we aim to reduce the burden related to participation in the septumtrial and minimize loss to follow-up. Individual subjects will not be replaced after withdrawal.

### Data management

Questionnaires will be administered, processed and stored digitally using the open source survey application LimeSurvey (LimeSurvey Project, Hamburg, Germany). Participant application forms (provided by otorhinolaryngologists in participating hospitals), medical history, and data on costs will be processed and stored using the data management system MACRO (InferMed, London, United Kingdom). All data will be handled according to the Dutch law (Dutch Data Protection Act). Data will be anonymized by a unique identification number.

### Statistical methods

#### Outcomes

Effects of surgery on health-related quality of life and nasal passage (rhinomanometry and peak nasal inspiratory flow) will be calculated as mean differences with 95 % confidence intervals. For health-related quality of life, short-term and long-term effects will be evaluated at 3, 6, 12, and 24 months follow-up, respectively. All analyses will be performed on an intention-to-treat basis. In addition to the intention-to-treat analysis, we will perform two sensitivity analyses: 1) a per-protocol analysis in which we will exclude adults in the watchful waiting group, who crossed-over to surgery, and 2) an “as treated” analysis in which we will add adults in the watchful waiting group, who crossed-over to surgery, to the septoplasty group. Potential modification of the effect of septoplasty will be evaluated using Poisson regression including interaction terms for gender, age, and severity of the septal deviation.

#### Cost-effectiveness analysis

As evidence regarding the cost-effectiveness of septoplasty is lacking, the main objective of the economic evaluation is to assess the balance between costs and effects of septoplasty as compared to watchful waiting for nasal obstruction in patients with a deviated nasal septum. The economic evaluation is based on the general principles of cost-effectiveness analysis (CEA) and will be performed alongside the randomized controlled trial. Considering the follow-up duration of 24 months, primary outcome measures for the economic evaluation will be costs (direct and indirect) and Quality-adjusted Life Years (QALY). The incremental cost-effectiveness ratio (ICER), i.e., cost per QALY gained, will be computed. Uncertainty will be determined using an appropriate method, such as bootstrapping, Taylor expansion, or the Fieller method. A cost-effectiveness acceptability curve will be derived, with the ability to evaluate efficiency by using different thresholds (willingness to pay) for 1 QALY. The impact of uncertainty surrounding deterministic parameters (e.g., prices) on the ICER will be explored using one-way sensitivity analyses on the range of extremes.

The cost analysis exists of two main parts. First, volumes of care will be measured prospectively on patient level using patient-based diaries and medical records. Patient-based diaries will record resource uses, such as doctor's visits, medication, hospital admissions, and surgical interventions, as well as out-of-pocket expenses such as costs for over-the-counter drugs. Additionally, traveling time to the outpatient clinic and related costs will be monitored using a diary. Where relevant, (missing) diary entries will be verified by data from medical records. Per arm (septoplasty and non-surgical management), full cost prices will be determined using activity-based costing. Productivity losses for patients will be estimated using a patient-based diary and interviews on a 3 to 12 months recall basis by the physician researcher. The friction cost-method will be applied following Dutch guidelines [30]. As second part of the cost analysis, cost prices for each volume of consumption will be determined. Cost prices will be multiplied with volumes recorded for each participant. Again, Dutch guidelines for cost analyses will be used [30]. Real cost prices will be determined in case no guidelines or standard prices are available for certain units of care or resources.

### Data monitoring

Participation in the septumtrial carries no risks additional to those associated with standard care. Therefore, no Data Monitoring Committee (DMC) is needed. However, the study is conducted in accordance with principles of Good Clinical Practice. For this reason we have developed a monitoring plan for data collection, aiming for:

1. Full monitoring of informed consents;
2. Full monitoring of serious adverse events (SAEs);
3. Monitoring of the first 20 patients at each follow-up visit, to ensure data quality of the following 180 patients.

### Harms

Septoplasty may lead to the following complications: nasal obstruction, nasal septal hematoma, nasal septal abscess, saddle nose or other nasal deformity, nasal septal perforation, epistaxis, acute or chronic rhinitis. All these complications are rare (occurring in less than 1 % of all cases) and most of them resolve without additional treatment. In selected cases, an additional intervention (drainage of hematoma or abscess, antibiotics, additional nasal packing, surgical correction of saddle nose or other nasal deformities) may be needed to resolve the complication. All adverse events (AEs) will be followed until they have abated, or until a stable situation has been reached. Depending on the event, follow-up may require additional tests or medical procedures as indicated, and/or referral to the general physician or a medical specialist. SAEs will be reported until the end of the study.

### Ethics and dissemination

#### Research ethics approval and amendments

The study protocol, informed consent form, and patient information brochure have been reviewed and approved by the sponsor (ZonMw, The Netherlands Organization for Health Research and Development) and the accredited medical ethics committee (Commission for Research in Human Subjects, in Dutch: Commissie Mensgebonden Onderzoek (CMO), region Arnhem – Nijmegen, the Netherlands). Since the enforcement of the Centrale Commissie Mensgebonden Onderzoek (CCMO) External Review Directive in 2012, local medical ethical committees of local participating centers are no longer involved in reviewing the study protocol of multicenter research in the Netherlands. Additional file 1 contains a list of all approved amendments and Additional file 2 contains the completed SPIRIT 2013 checklist, whereas Additional file 3 contains the informed consent form and patient information brochure (in Dutch). Additional file 4 provides an overview of the literature search described in the Background section.

#### Consent or assent

Potential participants will be informed by the otorhinolaryngologist and the physician researcher. Informed consent documents of all participants are obtained and co-signed by the physician researcher.

#### Dissemination policy

Results of the septoplasty trial will be communicated to participants, healthcare professionals and the public through newsletters, publications in peer-reviewed journals and presentations on national meetings of the Dutch ENT Society.

## Discussion

This ongoing pragmatic randomized controlled trial is the first study to assess the effectiveness of septoplasty compared to non-surgical management for nasal obstruction due to a deviated nasal septum in adults. So far, all available evidence for the effectiveness of septoplasty is based on observational studies. Observational studies, however, are known to have a high risk of bias. As a result, the beneficial effects of septoplasty that were reported in previous studies are possibly overestimated, since the improvement after surgery could also be explained by other factors, such as natural history or extraneous effects. To assess the effectiveness of interventions and to inform decisions about treatment options in clinical practice, a pragmatic randomized controlled trial remains the design of choice [31].

A major strength of our study is that the effectiveness of septoplasty is evaluated both in terms of subjective (health-related quality of life) as well as objective (nasal patency) outcome measures. Health-related quality of life is measured with four widely used questionnaires and nasal patency is assessed by two different methods (four-phase rhinomanometry and peak nasal inspiratory flow). Although subjective and objective outcome measures both provide valuable information about the effects of septoplasty on nasal obstruction, it is known that subjective and objective outcomes do not necessarily correlate [11]. We chose health-related quality of life as primary outcome measure to focus on the patient rather than the disease, as septoplasty aims at improving nasal symptoms and not merely straightening the nasal septum.

Another strength of our study is the relatively long duration of follow-up, i.e., 2 years for each participant. Follow-up visits will be scheduled at baseline and after 3, 6, 12, and 24 months. Most studies performed so far have a follow-up varying between 3 months and 1 year. This provides a distorted view of the effectiveness of surgery, as long-term results tend to differ from short-term outcomes, especially for turbinate surgery [32]. A follow-up of 2 years was chosen for this trial in order to evaluate whether short-term effects of surgery remain present after a longer period of time. Moreover, it provides insight in the number of patients wishing to cross-over from watchful waiting to surgery after the first year of follow-up.

Although necessary for a proper assessment of the effectiveness of septoplasty, the choice for a non-surgical control group bears the risk of hampering patient recruitment. Research has demonstrated that a potential barrier to trial participation is the possibility of being allocated to a placebo-only or active control intervention that is perceived to be less desirable than the study intervention [33]. Since eligible candidates need to have an indication to have septoplasty performed according to current medical practice, non-surgical management may be perceived as a less desirable treatment by both participants and otorhinolaryngologists. In our experience, a proper understanding of the rationale and aims of the study is of great importance to motivate trial participation.

At the time of submission of this trial protocol, 110 participants have been enrolled in the study. Patient recruitment will continue until 2016 to achieve the calculated sample size of 200 participants. Due to our experiences in previous ENT trials, we are confident that we will be able to meet the target sample size and to quantify the effects of septoplasty in a reliable and precise manner. With the results of this study we aim to contribute to the development of evidence-based guidelines regarding indications for septoplasty. As the impact of trial results upon policy and practice depends on their applicability, future research will focus on the representativeness of our trial sample to the population at large. However, loss of representativeness only affects generalizability of trial results if included patients differ from eligible but non-included patients with respect to determinants of the magnitude of treatment effect, i.e., effect modifiers [34]. In order to evaluate representativeness of our trial sample, we will conduct a future study in which baseline measurements (including demographic and disease-specific characteristics: e.g., type and severity of the nasal septal deviation) will be performed in all patients selected for septoplasty in a Dutch hospital not participating in the current trial. Moreover, we are currently collaborating with researchers from University College London, who have submitted an application for a comparable randomized controlled trial (RCT) on septoplasty for nasal obstruction, in response to a commissioned call from the Health Technology Assessment Programme of the National Institute for Health Research. Should this proposal be successful, we will continue working together for our shared objective of studying the effectiveness of septoplasty for nasal obstruction in adults with a deviated nasal septum.

## Trial status

Recruitment began in September 2013 and is currently ongoing. A total of 110 patients were enrolled by mid-July 2015.

## Additional files

Additional file 1:
**Administrative information in accordance with SPIRIT 2013 guidelines.** (PDF 85 kb) Additional file 2:
**SPIRIT 2013 checklist.** (PDF 118 kb) Additional file 3:
**Informed consent materials (in Dutch).** a) Informed consent form. b) Patient information brochure. (ZIP 633 kb) Additional file 4:
**Overview of literature search.** (PDF 11 kb)

## Abbreviations

AE: adverse event
C: contrast between interventions
CEA: cost-effectiveness analysis
CCMO: Centrale Commissie Mensgebonden Onderzoek
CMO: Commissie Mensgebonden Onderzoek
DARE: Database of Abstracts of Reviews of Effects
DMC: Data Monitoring Committee
ENT: ear, nose and throat
EQ-5D-3L: EuroQol-5 Dimensions-3 Levels
GBI: Glasgow Benefit Inventory
GHSI: Glasgow Health Status Inventory
HTA: Health Technology Assessment
I: interventions
ICER: incremental cost-effectiveness ratio
NHS EED: National Health Service Economic Evaluation Database
NOSE: Nasal Obstruction Symptom Evaluation
NSAIDs: non-steroidal anti-inflammatory drugs
NTR: Nederlands Trial Register (Dutch Trial Registry)
O: outcome measures
P: patient characteristics
QALY: Quality-adjusted Life Years
SAE: serious adverse event
SNOT-22: Sino-nasal Outcome Test-22
VAS: visual analogue scale

## Footnotes

1 For reasons of feasibility, in selected cases health-related quality of life questionnaires may be administered digitally to participants prior to the baseline visit. In case a subject decides to withdraw informed consent during the baseline visit, questionnaire derived data will be destroyed.
