# Supplementary material for: Effectiveness of septoplasty versus non-surgical management for nasal obstruction due to a deviated nasal septum in adults: study protocol for a randomized controlled trial
Source: Trials. 2015 Nov 4;16:500. doi: 10.1186/s13063-015-1031-4 (PMC4634847; doi:10.1186/s13063-015-1031-4)
Supplement: Additional file 1: — Administrative information in accordance with SPIRIT 2013 guidelines. (PDF 85 kb) [file 13063_2015_1031_MOESM1_ESM.pdf]

Administrative information in accordance with SPIRIT 2013 guidelines

Trial registration

a. **Registry**

Nederlands Trial Register / Dutch Trial Registry (www.trialregister.nl)

Registry name: Effectiveness of septoplasty

Trial identifier: NTR3868

Acronym: Septumtrial

b. **World Health Organization Trial Registration Data Set**

**Data category**	**Information**
Primary registry and trial identifying number	Trialregister.nl<br>NTR3868
Date of registration in primary registry	February 21, 2013
Secondary identifying numbers	U1111-1139-7254 WHO: The Universal Trial Number (UTN)
Source(s) of monetary or material support	ZonMw, The Netherlands Organization for Health Research and Development<br>Radboud university medical center Nijmegen, the Netherlands
Primary sponsor	Radboud university medical center Nijmegen, the Netherlands
Secondary sponsor(s)	ZonMw, The Netherlands Organization for Health Research and Development
Contact for public queries	Ms. C. Hendriks<br>Radboud university medical center<br>Department of Otorhinolaryngology<br>HP 377<br>PO Box 9101<br>6500 HB Nijmegen<br>The Netherlands<br>Email: Carine.Hendriks@radboudumc.nl<br>Phone: +31 (0)24 3610397<br>Fax: +31 (0)24 3540251
Contact for scientific queries	Dr. N. van Heerbeek<br>Radboud university medical center<br>Department of Otorhinolaryngology<br>HP 377<br>PO Box 9101<br>6500 HB Nijmegen

	The Netherlands<br>Email: Niels.vanHeerbeek@radboudumc.nl<br>Phone: +31 (0)24 3610397<br>Fax: +31 (0)24 3540251
Public title	Effectiveness of septoplasty
Scientific title	Effectiveness of septoplasty versus non-surgical management for nasal obstruction in adults with a deviated nasal septum: a multicenter, parallel-group, randomized controlled trial
Countries of recruitment	The Netherlands
Health condition(s) or problem(s) studied	Septoplasty, Deviated nasal septum, Nasal obstruction, Cost-effectiveness, Quality of life, Rhinomanometry
Intervention(s)	Intervention group: septoplasty, i.e. surgical correction of a deviated nasal septum according to current medical practice<br>Control group: non-surgical management, i.e. watchful waiting strategy or medical treatment such as steroids, antibiotics (intermittent and long-term), antihistamines, and analgesics
Key inclusion and exclusion criteria	Ages eligible for study: ≥18 years<br>Sexes eligible for study: both<br>Accepts healthy volunteers: no<br>Inclusion criteria: adult patients (≥ 18 years) selected for septoplasty according to current medical practice, i.e. symptomatic impairment of the nasal passage due to nasal septal deviation<br>Exclusion criteria:<br>1. Patients selected for septoplasty due to nasal septal perforation<br>2. Patients with previous septal surgery<br>3. Patients who undergo septoplasty as part of a cosmetic rhinoplasty procedure or in combination with spreader grafts<br>4. Patients with untreated allergic rhinitis or allergic rhinitis unresponsive to medical treatment
Study type	Interventional<br>Multicenter<br>Allocation: randomized<br>Intervention model: parallel assignment<br>Control: active

	Masking: none<br>Primary purpose: treatment
Date of first enrolment	September 2013
Target sample size	200
Recruitment status	Recruiting
Primary outcome(s)	Heath related quality of life measured with the validated Glasgow Benefit Inventory questionnaire and the Glasgow Health Status Inventory questionnaire
Key secondary outcomes	1. EQ-5D-3L<br>2. SNOT-22<br>3. Symptom scores measured with the Nasal Obstruction Symptom Evaluation (NOSE) Scale<br>4. Nasal patency measured with 4-phase rhinomanometry<br>5. Nasal patency measured with peak nasal inspiratory flow<br>6. Cost-effectiveness

Protocol version

Original protocol issue date: 14 March 2013

Authors: MvE, MR, CH, NvH

Amendments issue date: 8 October 2013

Protocol amendment number: 01 – 08

Authors: MvE, MR, CH, NvH

2013-Mar-14	Original
2013-Oct-8	Amendment 01 – 08<br>Amendment 01: Allowed the septoplasty to be combined with concurrent turbinate surgery, as approved by ZonMw and the medical ethics committee.<br>Amendment 02: Allowed the questionnaires to be administered digitally, as approved by the medical ethics committee.<br>Amendment 03: Allowed the ‘Glasgow Health Status Inventory’ to be added to the questionnaires, as approved by the medical ethics committee.

	Amendment 04: Allowed the physician researcher to use a checklist during the control visits of patients, as approved by the medical ethics committee.<br>Amendment 05: Allowed otorhinolaryngologists to use a standard operation form to report on the surgical procedure performed in trial patients, as approved by the medical ethics committee.<br>Amendment 06: Allowed patients to use a diary to report on costs made during the trial, as approved by the medical ethics committee.<br>Amendment 07: Allowed the email address of patients to be added on the inclusion form, in order to send out the questionnaires digitally, as approved by the medical ethics committee.<br>Amendment 08: Allowed the length and weight of patients to be added on the inclusion form, as approved by the medical ethics committee.

Funding

Non-industry funded trial. ZonMw (The Netherlands Organization for Health Research and Development) and Radboud university medical center Nijmegen are funding the costs of the septumtrial, covering budget for personnel and materials, as well as for implementation.

Roles and responsibilities

a. **Contributorship**

MR and NvH conceived the study and initiated the study design. MR and NvH are grant holders. MvE, MR, CH, and NvH drafted this manuscript. MR provided statistical expertise in clinical trial design. MvE will conduct data collection and will be responsible for randomization. MvE, NvH, and MR will participate in data analysis. All authors contributed to refinement of the study protocol and approved the final manuscript.

b. **Sponsor contact information**

Trial Sponsor: Radboud university medical center Nijmegen

Contact name: Mr. A. Verhoeven

Address:

Radboud university medical center

Department of Otorhinolaryngology

HP 377

PO Box 9101

6500 HB Nijmegen

The Netherlands

Telephone: +31 (0)24 3614450

Email: Arjan.Verhoeven@radboudumc.nl

c. Sponsor and funder

The funding sources will not have any role during the study's execution, analyses and interpretation of the data, nor in the decision to submit results.

d. Committees

Organizational structure and responsibilities:

Principal Investigators and Research Physician (Steering committee): NvH, MR, MvE

Design and conduct of the septumtrial

Preparation of protocol and revisions

Agreement of final protocol

Recruitment of patients

Reviewing progress of study

Publication of study reports

Members of TMC (Trial Management Committee)

Organizing Trial Management Committee meetings

Trial Management Committee (TMC): MvE, MR, CH, NvH

Weekly meetings

Study planning

Provide annual report to ZonMw and medical ethics committee

SAE reporting to Central Committee on Research Involving Human Subjects

Responsible for trial master file

Budget administration and contractual issues with individual centers

Data Management Committee: MvE, MR, CH, NvH, Michel van Kempen (CRCN), Hans Groenewoud (HEV)

Preparation of CRFs (Case Report Forms)

Maintenance of trial IT system and data entry

Data verification

Randomization

Lead Investigators

In each participating center a lead investigator (otorhinolaryngologist) will be identified, to be responsible for identification and recruitment of patients.
