# Supplementary material for: Effectiveness of septoplasty versus non-surgical management for nasal obstruction due to a deviated nasal septum in adults: study protocol for a randomized controlled trial
Source: Trials. 2015 Nov 4;16:500. doi: 10.1186/s13063-015-1031-4 (PMC4634847; doi:10.1186/s13063-015-1031-4)
Supplement: Additional file 3: — Informed consent materials (in Dutch). a) Informed consent form. b) Patient information brochure. (ZIP 633 kb) [file 13063_2015_1031_MOESM3_ESM.zip › 13063_2015_1031_MOESM3_ESM/Appendix IIIa Informed consent formR0.pdf]

Wat is het effect van een correctie van het neustussenschot op de kwaliteit van leven?

ToetsingOnline NL 4350309.091.13, versie 1/maart 2013

Ik heb de informatiebrief voor de proefpersoon gelezen. Ik kon aanvullende vragen stellen. Mijn vragen zijn genoeg beantwoord. Ik had genoeg tijd om te beslissen of ik meedoe.

Ik weet dat meedoen helemaal vrijwillig is. Ik weet dat ik op ieder moment kan beslissen om toch niet mee te doen. Daarvoor hoef ik geen reden te geven.

Ik geef toestemming om mijn huisarts te vertellen dat ik meedoe aan dit onderzoek.

Ik geef toestemming om de specialist(en) die mij behandelt te vertellen dat ik meedoe aan dit onderzoek.

Ik weet dat sommige mensen mijn gegevens kunnen zien. Die mensen staan vermeld in de Algemene brochure.

Ik geef toestemming om mijn gegevens te gebruiken, voor de doelen die in de informatiebrief staan.

Ik geef toestemming om mijn onderzoeksgegevens 15 jaar na afloop van dit onderzoek te bewaren.

Ik wil meedoen aan dit onderzoek.

Naam proefpersoon:

Handtekening: Datum : __ / __ / __

Ik verklaar hierbij dat ik deze proefpersoon volledig heb geïnformeerd over het genoemde onderzoek.

Als er tijdens het onderzoek informatie bekend wordt die de toestemming van de proefpersoon zou kunnen beïnvloeden, dan breng ik hem/haar daarvan tijdig op de hoogte.

Naam onderzoeker (of diens vertegenwoordiger):

Handtekening: Datum: __ / __ / __

Aanvullende informatie is gegeven door (indien van toepassing):

Naam:

Functie:

Handtekening: Datum: __ / __ / __

----

* Doorhalen wat niet van toepassing is.
