# Supplementary material for: Effectiveness of septoplasty versus non-surgical management for nasal obstruction due to a deviated nasal septum in adults: study protocol for a randomized controlled trial
Source: Trials. 2015 Nov 4;16:500. doi: 10.1186/s13063-015-1031-4 (PMC4634847; doi:10.1186/s13063-015-1031-4)
Supplement: Additional file 3: — Informed consent materials (in Dutch). a) Informed consent form. b) Patient information brochure. (ZIP 633 kb) [file 13063_2015_1031_MOESM3_ESM.zip › 13063_2015_1031_MOESM3_ESM/Appendix IIIb Patient information brochureR0.pdf]

Wat is het effect van een correctie van het neustussenschot op de kwaliteit van leven?

Informatie voor patiënten

Inleiding
Uw KNO-arts heeft u verteld over een onderzoek naar het effect van een correctie van het neustussenschot op de kwaliteit van leven. Om te beslissen of u aan dit onderzoek wilt meedoen, is goede voorlichting van onze kant nodig en een zorgvuldige afweging van uw kant. Daarom ontvangt u deze schriftelijke informatie. U kunt dit rustig (her)lezen en indien gewenst met uw naasten bespreken. Mocht u ondanks het lezen van deze folder nog vragen hebben, dan kunt u bellen met mevrouw C. Hendriks (projectcoördinator) via het telefoonnummer 024-3610397.

Neusverstopping en correctie van het neustussenschot
Uw KNO-arts heeft vastgesteld dat uw neusverstoppingsklachten waarschijnlijk veroorzaakt worden door een scheefstand van uw neustussenschot. Uw KNO-arts heeft aangegeven dat deze klachten waarschijnlijk te verhelpen zijn door uw neustussenschot recht te zetten. Behalve de voordelen van deze ingreep zijn ook de mogelijke nadelen besproken. Zo blijkt uit eerder onderzoek bij een deel van de patiënten de neuspassage ondanks een correctie van het neustussenschot niet te verbeteren. Daarnaast kan het voorkomen dat de neuspassage objectief wel verbetert, maar dat dit niet zo ervaren wordt. Met andere woorden, er passeert na de operatie wel meer lucht maar het voelt niet beter. De meest voor de hand liggende verklaring hiervoor is dat de verstoppingsklachten toch een andere oorzaak hebben.

Het onderzoek
Op dit moment staat niet vast wat het effect van een correctie van het neustussenschot op de kwaliteit van leven is en wie het meeste baat heeft bij een eventuele correctie van het neustussenschot. Om dit na te gaan wordt dit wetenschappelijk onderzoek uitgevoerd. Hierin onderzoeken we de kwaliteit van leven bij mensen met neusverstoppingsklachten ten gevolge van een scheefstaand neustussenschot en het eventuele effect dat een correctie van het neustussenschot daarop heeft. Daarnaast wordt de doorgankelijkheid van de neus gemeten en de mate waarin u gedurende de studieperiode klachten heeft van de neus en neuspassage.

Gang van zaken
Uw KNO-arts heeft u al informatie over het onderzoek gegeven en uw vragen beantwoord. Ook heeft hij/zij gevraagd of u interesse heeft in deelname. Als dit inderdaad zo is, ondertekent u een formulier waarmee u de KNO-arts toestemming geeft uw gegevens door te geven aan de medewerkers van het onderzoek in het Radboud universitair medisch centrum te Nijmegen. Zij zullen telefonisch met u contact opnemen en aanvullende informatie geven. Ook zullen zij nagaan of u voldoet aan de criteria om mee te doen met het onderzoek. Indien u op basis van deze informatie besluit deel te nemen, vragen we u een toestemmingsformulier te ondertekenen. De arts ondertekent dit formulier ook. Vervolgens wordt er een controlebezoek afgesproken. Dit bezoek duurt in totaal 30 minuten. Tijdens dit bezoek wordt het volgende aanvullende onderzoek gedaan:
- doornemen van verschillende vragenlijsten
- onderzoek van de keel, neus en oren (algemeen KNO-onderzoek)
- rhinomanometrie (meten van de doorgankelijkheid van de neus)

Het algemeen KNO-onderzoek en de rhinomanometrie zijn beide niet belastende of pijnlijke onderzoeken. Hierna wordt tijdens dit bezoek door loting bepaald welke behandeling u krijgt (een correctie van het neustussenschot of een afwachtend beleid zonder operatie). Dit geven wij door aan uw KNO-arts. Daarnaast vragen we u gedurende de studie een dagboekje bij te houden waarin klachten van de neus vermeld kunnen worden en eventuele andere behandelingen (neussprays, zoutspoelingen, neusklemmetje, andere operaties, e.d.).
Hierna vinden nog vier controlebezoeken plaats, te weten 3, 6, 12 en 24 maanden later. Tijdens deze controlebezoeken wordt het hierboven beschreven aanvullende onderzoek herhaald.

Voor- en nadelen
Deelname aan dit onderzoek betekent voor u een extra tijdsinspanning. Omdat het niet bekend is of en bij wie een correctie van het neustussenschot zinvol is, is momenteel niet van tevoren in te schatten bij wie een correctie van het neustussenschot een groot effect heeft en bij wie geen of een kleiner effect. Door mee te doen aan de studie draagt u bij aan het verbeteren van de kennis hierover en het daarmee verbeteren van de

zorg voor toekomstige patiënten. Indien u door loting in de groep met patiënten komt die geopereerd worden, wordt er niets anders gedaan behoudens de extra bezoeken dan wanneer u niet mee zou doen aan de studie (met andere woorden, u wordt gewoon geopereerd). Komt u in de groep die niet geopereerd wordt, dan kan achteraf blijken dat een operatie inderdaad voor u ook niet de beste optie was en blijft een operatie u bespaard. Ook kan na de studie als dat nodig is alsnog een correctie van het neustussenschot uitgevoerd worden.

Opereren of niet

Omdat er door loting wordt bepaald welke patiënten worden geopereerd en bij welke patiënten wordt afgewacht, kunnen wij niet van tevoren zeggen of een operatie bij u wel of niet zal plaatsvinden. Het effect van een operatie op uw klachten is echter niet te voorspellen. Met andere woorden, we weten op voorhand ook niet waar u meer baat bij zult hebben: opereren of afwachten. Indien u desondanks een sterke voorkeur heeft voor een operatie, is het verstandig om dit te bespreken met uw KNO-arts of met de arts-onderzoeker. In dat geval kunt u ervoor kiezen af te zien van deelname aan de studie.

Toestemming

Ook als u toestemming heeft gegeven, staat het u vrij deze toestemming te allen tijde weer in te trekken. U hoeft daar geen reden voor op te geven. Dit zal geen gevolgen hebben voor uw verdere behandeling. Alle informatie die in het kader van het onderzoek verzameld wordt, zal anoniem en vertrouwelijk verwerkt worden.

Verzekering en vergoeding

De onderzoekers hebben van de METC (Medisch Ethische Toetsingscommissie), verbonden aan het Radboud universitair medisch centrum, ontheffing gekregen van de plicht om een speciale proefpersonenverzekering af te sluiten. Deze ontheffing is verleend omdat er voor proefpersonen geen risico's zijn verbonden aan deelname aan het onderzoek.
Aan het onderzoek zijn voor u geen extra kosten verbonden. Uw reguliere bezoeken aan de KNO-arts worden gedeclareerd bij uw verzekeraar en indien van toepassing verrekend met uw eigen risico. Voor de kosten van de operatie geldt, indien deze plaatsvindt, hetzelfde. Aan de extra 2-3 bezoeken die in het kader van het onderzoek plaatsvinden, zijn voor u noch voor uw verzekeraar kosten verbonden.

Organisatie, uitvoering en financiering van het onderzoek

Het onderzoek wordt georganiseerd door de afdeling KNO-heelkunde, de afdeling Operatiekamers en de afdeling Epidemiologie, Biostatistiek en HTA van het Radboud universitair medisch centrum te Nijmegen. Voor het onderzoek is goedkeuring verkregen van de METC (Medisch Ethische Toetsingscommissie) van het Radboud universitair medisch centrum. Het project wordt uitgevoerd in de KNO-praktijken van verschillende ziekenhuizen in Nederland. Dit onderzoek wordt gefinancierd door het programma DoelmatigheidsOnderzoek van ZonMw.

Onafhankelijke arts en wat te doen bij klachten

Indien u wilt overleggen met een onafhankelijke arts, die niet bij de uitvoering van het onderzoek betrokken is, kunt u contact opnemen met Dr. G. van den Broek, KNO-arts in het Radboud universitair medisch centrum, telefoonnummer 024-3613508. Als u klachten heeft over het onderzoek, kunt u dit melden aan de medewerkers van het onderzoek of uw behandelend KNO-arts. Als u dit liever niet wilt, dan kunt u contact opnemen met het Voorlichtingscentrum van het Radboud universitair medisch centrum (telefoonnummer 024-3617000).

Inlichtingen

Als u vragen heeft over het onderzoek, aarzel dan niet contact op te nemen met de medewerkers van het onderzoek in het Radboud universitair medisch centrum:
Drs. M.van Egmond, arts-onderzoeker
Mw. C. Hendriks, projectcoördinator
Dr. N. van Heerbeek, KNO-arts, projectleider
Prof. Dr. M. Rovers, hoogleraar Evidence Based Surgery, medeprojectleider
Tel: 024-3610397
E-mail: Carine.Hendriks@radboudumc.nl

Radboudumc

Toestemmingsverklaring

Door het onderteken van dit formulier ga ik ermee akkoord dat, in het kader van het doelmatigheidsonderzoek naar correcties van het neustussenschot, mijn gegevens worden doorgegeven aan de onderzoekers van het Radboud universitair medisch centrum.
De onderzoekers mogen na ontvangst van de gegevens contact met mij opnemen om eventuele vragen te beantwoorden en afspraken over het onderzoek te maken.

Naam: ………………………………………………………………………………………………………

Adres: ………………………………………………………………………………………………………

Postcode: ……………………………………………………………………………………………………

Plaats: ………………………………………………………………………………………………………

Telefoonnummer: …………………………………………………………………………………………

Geboortedatum: ……………………………………………………………………………………………

Ik ben onder behandeling van dr: ………………………………………………………………..

Naam ziekenhuis: ………………………………………………………………………………………

Plaats ziekenhuis: ………………………………………………………………………………………

Datum + Handtekening: ………………………………………………………………………….

Na ondertekening dit formulier s.v.p. opsturen aan:

Radboudumc
377 – KNO C. Hendriks
Postbus 9101
6500 HB NIJMEGEN
