# Supplementary material for: Effectiveness of septoplasty versus non-surgical management for nasal obstruction due to a deviated nasal septum in adults: study protocol for a randomized controlled trial
Source: Trials. 2015 Nov 4;16:500. doi: 10.1186/s13063-015-1031-4 (PMC4634847; doi:10.1186/s13063-015-1031-4)
Supplement: Additional file 4: — Overview of literature search. (PDF 11 kb) [file 13063_2015_1031_MOESM4_ESM.pdf]

Appendix IV: Overview of literature search for systematic reviews or randomized controlled trials on the effectiveness of septoplasty

**Database**	**Search terms**	**Hits**
PubMed	(SEPTOPLASTY[Title/Abstract]) OR (septal surgery[Title/Abstract])) AND ((clinical[Title/Abstract] AND trial[Title/Abstract]) OR clinical trials[MeSH Terms] OR clinical trial[Publication Type] OR random*[Title/Abstract] OR random allocation[MeSH Terms] OR therapeutic use[MeSH Subheading])	255
Cochrane Library	Septoplasty	1
Current Controlled Trials	Septoplasty	18
ClinicalTrials.gov	Septoplasty	7
DARE, NHS EED, and HTA databases	Septoplasty	6
**Total number of hits**		**287**
Exclusion criteria	- Duplicates<br>- Other interventions studied (no septoplasty versus watchful waiting)<br>- Descriptive reviews (instead of systematic reviews)<br>- No trial	4<br>91<br><br>3<br>189
**Total number of excluded studies**		**287**
**Total number of included studies**		**0**
